# Effects of Short-Term Fasting on mRNA Expression of Ghrelin and the Peptide Transporters PepT1 and 2 in Atlantic Salmon (*Salmo salar*)

**DOI:** 10.3389/fphys.2021.666670

**Published:** 2021-06-21

**Authors:** Gianmarco Del Vecchio, Floriana Lai, Ana S. Gomes, Tiziano Verri, Tharmini Kalananthan, Amilcare Barca, Sigurd Handeland, Ivar Rønnestad

**Affiliations:** ^1^Laboratory of Applied Physiology, Department of Biological and Environmental Sciences and Technologies, University of Salento, Lecce, Italy; ^2^Department of Biological Sciences, University of Bergen, Bergen, Norway

**Keywords:** ghrelin, peptide transporters, slc15, fasting, digestive tract, fish

## Abstract

Food intake is a vital process that supplies necessary energy and essential nutrients to the body. Information regarding luminal composition in the gastrointestinal tract (GIT) collected through mechanical and nutrient sensing mechanisms are generally conveyed, in both mammals and fish, to the hypothalamic neurocircuits. In this context, ghrelin, the only known hormone with an orexigenic action, and the intestinal peptide transporters 1 and 2, involved in absorption of dietary di- and tripeptides, exert important and also integrated roles for the nutrient uptake. Together, both are potentially involved in signaling pathways that control food intake originating from different segments of the GIT. However, little is known about the role of different paralogs and their response to fasting. Therefore, after 3 weeks of acclimatization, 12 Atlantic salmon (*Salmo salar*) post-smolt were fasted for 4 days to explore the gastrointestinal response in comparison with fed control (*n* = 12). The analysis covered morphometric (weight, length, condition factor, and wet content/weight fish %), molecular (gene expression variations), and correlation analyses. Such short-term fasting is a common and recommended practice used prior to any handling in commercial culture of the species. There were no statistical differences in length and weight but a significant lower condition factor in the fasted group. Transcriptional analysis along the gastrointestinal segments revealed a tendency of downregulation for both paralogous genes *slc15a1a* and *slc15a1b* and with significant lowered levels in the pyloric ceca for *slc15a1a* and in the pyloric ceca and midgut for *slc15a1b*. No differences were found for *slc15a2a* and *slc15a2b* (except a higher expression of the fasted group in the anterior midgut), supporting different roles for *slc15* paralogs. This represents the first report on the effects of fasting on *slc15a2* expressed in GIT in teleosts. Transcriptional analysis of ghrelin splicing variants (*ghrl-1* and *ghrl-2*) showed no difference between treatments. However, correlation analysis showed that the mRNA expression for all genes (restricted to segment with the highest levels) were affected by the residual luminal content. Overall, the results show minimal effects of 4 days of induced fasting in Atlantic salmon, suggesting that more time is needed to initiate a large GIT response.

## Introduction

Food intake is an important mechanism that allows for acquiring all necessary energy and essential nutrients for subsistence, activity, and growth (Schwartz et al., 2000). In this process, the gastrointestinal tract (GIT) serves a key role, being constantly exposed to vastly different chemical substances, ions, micro-/macronutrients, and microorganisms. The regulation of the digestive and absorption processes is based on information about luminal content and filling, which are first conveyed through nutrient sensing and stretch sensory mechanisms that underlie the initiation of neuronal and humoral signals (Rønnestad et al., 2014, 2017; Daniel and Zietek, 2015; Zietek and Daniel, 2015; Conde-Sieira and Soengas, 2017) and then sent to the main regulatory centers of food intake, feeding behavior, and energy expenditure in the hypothalamic neurocircuits (both for mammals and fish) (Cerdá-Reverter and Canosa, 2009; Blouet and Schwartz, 2010; Morton et al., 2014), through orexigenic and anorexigenic neuropeptides (Volkoff, 2016; Conde-Sieira and Soengas, 2017; Delgado et al., 2017; Rønnestad et al., 2017).

Ghrelin (GHRL) is a hormone secreted mainly in the stomach and the only known to have a stimulating-appetite action (orexigenic) in all the vertebrate systems (Higgins et al., 2007; Patton and Mistlberger, 2013). In fish, Ghrl is mainly secreted in the stomach, as in mammals; however, for stomach-less species, such as the cyprinids goldfish (*Carassius auratus*) and zebrafish (*Danio rerio*), early studies confirmed the intestine to be the major site of *ghrl* expression and release (Unniappan et al., 2002; Olsson et al., 2008). Further to this, a recent study has shown that among agastric fish species, the *ghrl* gene is only found in the cyprinid family (Lie et al., 2018), which means that several of the known model teleost species like medaka (*Oryzias latipes*) and fugu (*Takifugu rubripes*) lack the gene coding for Ghrl. Comparative studies among fish species suggest that the generalized orexigenic role of Ghrl in appetite and food intake is not as clear as for mammals. In fact, anorexigenic and contradictory effects have been assessed in some species, such as Mozambique tilapia (*Oreochromis mossambicus*) (Peddu et al., 2009), rainbow trout (*Oncorhynchus mykiss*) (Jönsson et al., 2010; Velasco et al.,2016a,b), and Atlantic salmon (*Salmo salar*) (Murashita et al., 2009; Hevrøy et al., 2011; Vikesa et al., 2015), suggesting a probable species- and form-specific Ghrl role in the regulation of feeding and metabolism in fish (Volkoff, 2016).

During digestion of dietary proteins, a multitude of peptides of different sizes and composition can be found, and the intestinal peptide transporter 1 (PEPT1), also known as SoLute carrier family 15 member a1 (*SLC15A1*), serves a key role in the uptake of dietary amino acids in di- and tripeptide forms in all the vertebrates (Daniel, 2004; Smith et al., 2013a). Several of these absorbed peptides have been shown to have also a bioactive function in the complex mechanism of nutrient sensing and regulation of dietary metabolism (Rønnestad et al., 2014; Daniel and Zietek, 2015; Zietek and Daniel, 2015), suggesting that the PEPT1 is also involved in the gut–brain signaling axis. The peptide transporter has been identified and characterized in many vertebrate species, and lately, much attention has also been placed in lower vertebrates, teleost species included, where less is known. The studied fish species include zebrafish (Verri et al., 2003), Atlantic cod (*Gadus morhua*) (Rønnestad et al., 2007), European sea bass (*Dicentrarchus labrax*) (Sangaletti et al., 2009), Atlantic salmon (Rønnestad et al., 2010), Antarctic Icefish (*Chionodraco hamatus*) (Rizzello et al., 2013), and Grass Carp (*Ctenopharyngodon idella*) (Liu et al., 2013). The increased availability of genome sequences for teleosts has progressively clarified the co-presence of two transporters, as a result of an additional teleost-specific whole-genome duplication (WGD) event (Gonçalves et al., 2007; Bucking and Schulte, 2012; Con et al., 2017; Orozco et al., 2017; Chourasia et al., 2018). The two transporters are called Pept1a, expressed by *slc15a1a* gene, and Pept1b, expressed by *slc15a1b* gene, respectively. The emerging *slc15a1a* paralog has just recently been identified, and therefore, limited information is still available for this gene. So far, only two studies on teleost species are found, i.e., in zebrafish (Vacca et al., 2019) and in Atlantic salmon (Gomes et al., 2020). Consequently, the largest part of the data available so far on teleost species about the functional role of Pept1 proteins refers to the Pept1b type (Verri et al., 2010; Romano et al., 2014; Verri et al., 2017).

The intestinal peptide transporter peptide transporter 2 (PEPT2) is another plasma membrane transporter of di- and tripeptides belonging to the same Solute carrier family 15 (member a2) as PEPT1. PEPT2 is well characterized in mammals, where high expressions are found in the kidney, peripheral, and central nervous system, lung (Rubio-Aliaga and Daniel, 2002, 2008; Daniel and Rubio-Aliaga, 2003; Kamal et al., 2008), and GIT, where it seems to be not expressed by epithelial cells but, rather, by the enteric glial cells and tissue-resident macrophages (Rühl et al., 2005). As for Pept1, compared to mammals and higher vertebrates, there is little knowledge about this transporter in lower vertebrates. In teleost species, Pept2 has been, in fact, characterized only in zebrafish (Romano et al., 2006), whereas no data are available in Atlantic salmon so far.

Thus, based on the available information, it is likely that both Ghrl and Pept1 and/or Pept2 may be involved in signaling the quantitative and qualitative presence of nutrients in different sections of the GIT. Moreover, due to both teleost-specific and later salmonid WGD events (Davidson et al., 2010; Berthelot et al., 2014; Macqueen and Johnston, 2014; Pasquier et al., 2016), Atlantic salmon has several paralogs of these genes and, therefore, identifying any probable functional evolutionary adaptations of paralogs is also important. In fish, Pept1 represents an important part of the absorptive capacity of protein nitrogen for the animal, especially for teleosts, where it plays an important role in animal growth (Verri et al., 2011). Its messenger RNA (mRNA) expression is known to change in response to a fasting period: reduced expressions (for both paralogous genes) are found in 7 days fasted Nile tilapia (*Oreochromis niloticus*) (Huang et al., 2015), in both short- and long-term fasted zebrafish (Tian et al., 2015), in 6 days fasted juvenile Atlantic salmon (Rønnestad et al., 2010), and in sea bass (*Dicentrarxus labrax*) (Terova et al., 2009). The peptide hormone Ghrl has received attention in aquaculture since stimulation of food intake is important, particularly since fish meal has been replaced with plant-based dietary ingredients and the palatability reduced (Kousoulaki et al., 2013). Ghrl has shown orexigenic effects in induced fasting goldfish (Kang et al., 2011; Nisembaum et al., 2014) and in Nile tilapia (Schwandt et al., 2010), whereas low Ghrl plasma levels and contradictory effects are found in fasted Atlantic salmon (Hevrøy et al., 2011).

In the Atlantic salmon aquaculture production, a period of 2–4 days of fasting is a common and recommended practice prior to any handling, transportation, and harvest in order to allow the complete evacuation of the gut to minimize impacts on stress, metabolism, fish welfare, and even mortality (Waagbø et al., 2017). Moreover, fasting also ensures proper hygiene after harvest (Einen et al., 1998; Robb, 2008). To date, analysis on metabolic rate, stress response, and morphometric parameters on Atlantic salmon postsmolts has assessed that a fasting period up to 4 weeks has no negligible effects on salmon welfare (Hvas et al., 2020). To learn more about the GIT properties and how it responds to a standard fasting period, the effects of 4 days of induced fasting on morphometric parameters (length, weight, condition factor, and status of GIT digesta content) and on the transcriptional changes (ghrelin splicing variants and peptide transporters paralogs) along the GIT segments were evaluated. Moreover, how the presence/absence of the digesta in the GIT affects the mRNA expression of the investigated genes through correlation analysis was also assessed.

## Materials and Methods

### Ethics Statement

The experiment and sampling were conducted in accordance with Norwegian Animal Research Authority regulations and were approved by local representative of Animal Welfare at the Department of Biological Sciences, University of Bergen (Norway).

### Experimental Design and Sampling

This study is part of a larger experiment where the main data on fish growth and performance were assessed (Kalananthan et al., 2020a). A subgroup of fish was used to evaluate the transcriptional changes of ghrelin splicing variants and peptide transporters paralogs along the GIT segments. In brief, Atlantic salmon postsmolt (ca. 250 g; *n* = 96) obtained from Engesund farm (Fitjar, Norway) were randomly distributed into two 2,000-L tanks (48 fish per tank) at the Industrial Lab fish facility (ILAB, Bergen, Norway). The rearing tanks were connected to a flow through system at 10°C with a salinity of 27 ppt, water flow of 16 L/min, oxygen saturation always above 80%, and under constant light (all parameters checked daily). Fish were fed to satiety with commercial dry feed pellets (Biomar intro 75 HH 50 mg Q) using an automatic feeder throughout 24 h. After 3 weeks of acclimatization, the two tanks were randomly labeled into two experimental groups; one tank was kept as control (fed group), i.e., fish fed normally as described above, whereas the other tank was kept as treated (fasted group) 21 fish per tank (263 ± 13.06 and 275.7 ± 15.68 g; mean ± SEM) were sampled as a baseline control. After 4 days, 12 fish were sampled from the fed group (281.41 ± 25.20 g; mean ± SEM) and 12 from the fasted group (256.66 ± 21.14 g, mean ± SEM) using a lethal dose of 200 mg/L of MS222 (tricaine methanesulfonate, Scan-Vacc, Hvam, Norway). Length (*L*) and weight (*W*) were recorded and used to calculate Fulton’s condition factor (*K*) using the following equation (Froese, 2006):

$$K=100\,\frac{W}{L^{3}}$$

where *L* is expressed in cm and *W* in g.

Gastrointestinal tract was carefully, but rapidly, dissected by cutting at the extremity of the esophagus and hindgut (HG), using surgical clamps to avoid loss of content. Stomach (ST), anterior midgut (AMG), midgut (MG), and HG compartments were separated by surgical clamps and, then, carefully opened in order to collect and weight the inner content of each segment (see Supplementary Figure 1). Next, tissue samples of the stomach [two samples: anterior stomach (ASt) and posterior stomach (PSt)], pyloric ceca (PC), anterior midgut (AMG), MG and HG [two samples: anterior hindgut (AHG) and posterior hindgut (PHG)] were collected, transferred into RNAlater solution (Invitrogen, Carlsbad, CA, United States), kept at 4°C overnight, and then stored in –80°C until further analysis.

### Total RNA Extraction and cDNA Synthesis

Total RNA was isolated using TRI reagent (Sigma-Aldrich, St. Louis, MO, United States) according to the manufacturer’s instructions. To avoid any remnants of genomic DNA, 10 μg of total RNA samples were treated with TURBO DNase-free Kit (Ambion Applied Biosystems, Foster City, CA, United States) following the manufacturer’s instructions. A NanoDrop ND-1000 spectrophotometer (Thermo Fisher Scientific, Waltham, MA, United States) and an Agilent 2100 Bioanalyzer (Agilent Technologies, Sta Clara, CA, United States) were used to evaluate the quantity (ng/μl) and purity and quality (RNA integrity number, RIN) of the total RNA extracted, respectively. Only RNAs with RIN values >6 were used for further analysis. First-strand complementary DNA (cDNA) was synthetized from 2 μg of DNase-treated total RNA using SuperScript III First-Strand Synthesis kit (Invitrogen, Carlsbad, CA, United States) according to the manufacture’s protocol.

### Primer Design and RT-qPCR

Specific primers for all the target genes (*slc15a1a*, *slc15a1b*, *slc15a2a*, *slc15a2b*, *ghrl-1*, and *ghrl-2*) were designed spanning an exon–exon junction when possible (Table 1). All the designed primers were tested for cycle of quantification (Cq), primers efficiency (E), and melting peaks. Next, reverse transcription quantitative PCR (RT-qPCR) products were resolved in a 2% agarose gel, purified using E.Z.N.A. Gel Extraction Kit (Omega Bio-Tek, Norcross, GA, United States) and cloned into a pCR4-TOPO vector (Thermo Fisher Scientific, Waltham, MA, United States). Sequencing was performed at the University of Bergen Sequencing Facility (Bergen, Norway) and sequences identity confirmed using blastn analysis against the Atlantic salmon genome GenBank database.

**TABLE 1:** Primer sequences for reverse transcription quantitative PCR (RT-qPCR) expression analysis in Atlantic salmon.

Gene	GenBank Acc. No.	Primer sequences 5′–3′	Amplicon (bp)	*R*^2^	Efficiency (%)
*slc15a1b*	NM_001146682	**F:** GCCGTGGGCAACATCATAGT **R:** CTGGGTCCGTGTAGGTGTAGAA	148	0.999	93.91
*slc15a1a*	XM_014172951	**F:** TTCCCTCAAACCTCAGTGCC **R:** CCGCTCCAGCCATACCATTA	108	0.9982	94.41
*slc15a2a*	XM_014165384	**F:** GGGGGACACAACAAGACCAT **R:** CCGCGTGTTTATGAACCTCA	198	0.9943	94.42
*slc15a2b*	XM_014173652	**F:** GGATCAGTGGATGGAGTTCCTG **R:** TCTGGTTCTTTTTCAAGTTGTCTTC	170	0.9999	96.26
*ghrl-1*	NM_001142709.1	**F:** CCAGAAACCACAGGTAAGACAGGGTA **R:** GAGCCTTGATTGTATTGTGTTTGTCT	128	0.9999	91.5
*ghrl-2*	NM_001139585.1	**F:** TCCCAGAAACCACAGGGTAAA **R:** GAGCCTTGATTGTATTGTGTTTGTCT	121	0.999	94.32
*s20*	NM_001140843.1/XM_014180600.1 (splice variant)/XM_014157862.1	**F:** GCAGACCTTATCCGTGGAGCTA **R:** TGGTGATGCGCAGAGTCTTG	84	0.9951	92.36

*The accession number is indicated for each investigated gene. The amplicon size (bp), *R*^2^, and RT-qPCR efficiency (%) are indicated for each primer pair.*

Standard curves for each target and reference gene ribosomal protein s20 (*s20*) were generated from the gene cloned in pCR4-TOPO vector using a 10-fold serial dilution. All the RT-qPCR reactions were performed in duplicate using iTaq Universal SYBR Green Supermix (Bio-Rad, Hercules, CA, United States) in a 20-μl final reaction volume. The following RT-qPCR protocol was performed: (1) 95°C for 30 s, (2) 95°C for 5 s, (3) 60°C for 25 s (steps 2–4 repeated for 40 cycles) in a CFX96 Real-Time System (Bio-Rad Laboratories, Hercules, CA, United States) in connection to CFX Manager Software version 3.1 (Bio-Rad, Laboratories, Hercules, CA, United States). Melting curve analysis over a range of 65–95°C (increment of 0.5°C for 2 s) allowed for the detection of possible nonspecific products and/or primer dimers. Finally, absolute quantification of mRNA expression level was calculated for each gene using the respective standard curve and following the equation:

$$\mathrm{Copy}\,\mathrm{number}=10^{\frac{C\,q-i\,n\,t\,e\,r\,c\,e\,p\,t}{s\,l\,o\,p\,e}}$$

The copy number was divided by the nanogram of total RNA used for each target gene. The copy number/ng of total RNA of the reference gene *s20* was used to normalize the RT-qPCR data.

### Statistical Analysis

All the morphometric (*L*, *W*, *K*, and wet content/fish weight %) and mRNA expression (*ghrl-1*, *ghrl-2, slc15a1a*, *slc15a1b*, *slc15a2a*, and *slc15a2b*) data were tested for normality and equal variance using D’Agostino-Pearson test and *F*-test ratio. Grubb’s outlier test was run prior to statistical evaluations. To achieve normal distribution, gene expression data were log-transformed, and the analysis of differential expression between the fed and fasted groups was performed with two-tailed *t*-test. When either the *F*-test or the normality test failed, the nonparametric Mann–Whitney test was performed. For ghrelin splice variants, to assess eventual effects of stomach segments and/or presence/absence of food, a two-way ANOVA test, followed by a Sidak’s multiple comparisons test was performed. All the statistical analyses and the graphs were produced using GraphPad Prism 9.1.0 (GraphPad Software, La Jolla, CA, United States). For correlation analysis data, exploration was first performed to identify possible outliers and collinearity, and subsequent analysis was restricted to tissues with relevant mRNA expression levels. Correlation analysis between mRNA expression of target genes and the content in GIT sections was analyzed using generalized linear models (GLMs). Because gene expression was best described by a log-normal distribution, log-transformed gene expression was modeled as a function of tissue-specific content. The model selection procedure was elaborated as follows: (1) all the GIT section contents were tested for each gene, and the region that explained gene expression best was selected (i.e., models with content in ST, AMG, MG, and HG as covariate were compared and selected based on Akaike information criterion); (2) the best model was used to analyze the relationship between content of GIT section and gene expression. To account for different locations of expression, an interaction term between the location of expression and GIT section content was added. This allowed for different relationships between gut content and expression in different tissues, i.e., it represents a common model for each gene with shared intercept for all tissues but different slopes. A graphic depiction of the fitted model was created. Analyses were performed in R 4.0.2 (R Core Team, 2020) using Tidyverse packages (Wickham et al., 2019).

For all tests, a *p* < 0.05 was considered significant (^∗^*p* < 0.05; ^∗∗^*p* < 0.01; ^∗∗∗^*p* < 0.001). All data are presented as mean ± SEM, unless otherwise stated.

## Results

### Morphometric and Gastrointestinal Fullness Analysis

The morphometric analysis after 4 days of fasting revealed no statistical differences in *W* (g) and *L* (cm) between the two groups. Conversely, the *K* was significantly lower (*p* < 0.05) in the fasted group (1.002 ± 0.015 g/cm^3^) compared to the fed group (1.083 ± 0.029 g/cm^3^). The wet content/fish weight % in each GIT segment was significantly lower in the fasted group in ST (empty stomach vs. 0.274 ± 0.007; *p* < 0.0001), AMG (0.104 ± 0.01 vs. 0.498 ± 0.04; *p* < 0.0001), MG (0.042 ± 0.01 vs. 0.265 ± 0.03; *p* < 0.001), and HG (0.054 ± 0.01 vs. 0.316 ± 0.03; *p* < 0.0001).

### Effects of 4 Days of Fasting on mRNA Expression Levels

#### Di- and Tripeptide Transporters (*slc15a1a*, *slc15a1b*, *slc15a2a*, and *slc15a2b*)

**FIGURE 1:**
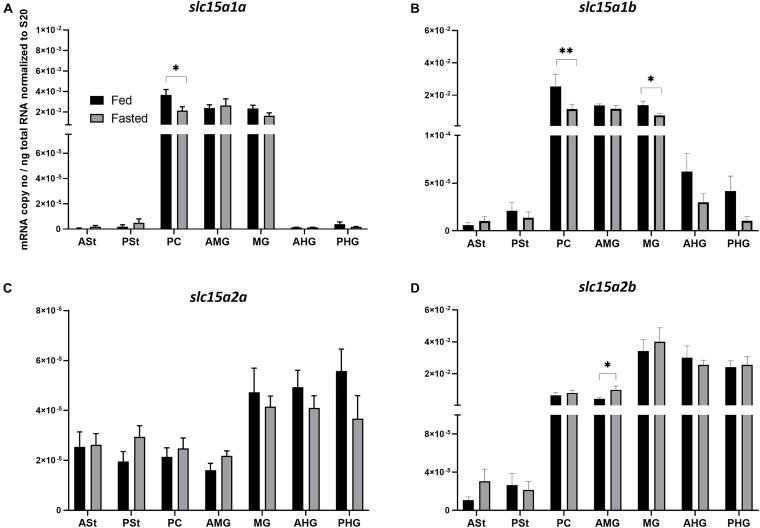
Comparison of mRNA expression levels of **(A)** *slc15a1a*, **(B)** *slc15a1b*, **(C)** *slc15a2a*, and **(D)** *slc15a2b* between fed (control) and 4-day fasted groups along the gastrointestinal tract. **(A)** Data are presented as mean ± SE [*n* = 12 per group, except for PSt fed and fasted, PC fed, AMG fed, MG fed, and AHG fed (*n* = 11); ASt fasted (*n* = 9) and ASt fed (*n* = 8)] of the normalized expression using the reference gene *s20*. **(B)** Data are presented as mean ± SE [*n* = 12 per group, except for ASt fed, PSt fed, PC fasted, MG fed and fasted and PHG fasted (*n* = 11), and AMG fed (*n* = 10)] of the normalized expression using the reference gene *s20*. **(C)** Data are presented as mean ± SE [*n* = 12 per group, except for PSt fed and fasted, PC fed and fasted, AMG fasted and PHG fasted (*n* = 11), and AMG fed (*n* = 10)] of the normalized expression using the reference gene *s20*. **(D)** Data are presented as mean ± SE [*n* = 12 per group, except for PSt fed, AMG fed, MG fasted, AHG fasted and PHG fed and fasted (*n* = 11), and PC fasted (*n* = 10)] of the normalized expression using the reference gene *s20*. Statistical analysis: unpaired *t*-test (*p < 0.05; **p < 0.01). ASt, anterior stomach; PSt, posterior stomach; PC, pyloric ceca; AMG, anterior midgut; MG, midgut; AHG, anterior hindgut; PHG, posterior hindgut.

The analysis of mRNA expression levels of the di- and tripeptide transporters along the GIT of Atlantic salmon revealed a similar trend for both *slc15a1a* and *slc15a1b* (Figures 1A,B). In both cases, the highest expression levels were observed in PC, AMG, and MG, with *slc15a1b* (Figure 1B) always showing much higher mRNA expression levels than *slc15a1a* (Figure 1A). Fasting induced a significantly (*p* < 0.05) downregulation in the PC for *slc15a1a* and in PC (*p* < 0.01) and MG (*p* < 0.05) for *slc15a1b*. Peptide transporters 2 types (*slc15a2a* and *slc15a2b*) showed different trends of expression along the GIT (Figures 1C,D). While *slc15a2a* (Figure 1C) showed a similar low expression level, the paralog *slc15a2b* (Figure 1D) revealed a progressive increase in expression along the GIT segments. The paralog *slc15a2b* showed much higher expression levels compared to the paralogous gene *slc15a2a*. Fasting induced a significantly upregulation in the AMG for *slc15a2b* (*p* < 0.05). Furthermore, as for *slc15a1*, 4 days of fasting induced a tendency of downregulation in *slc15a2b*, but in this case, no statistical differences were found.

#### Ghrelin (*ghrl-1* and *ghrl-2)*

**FIGURE 2:**
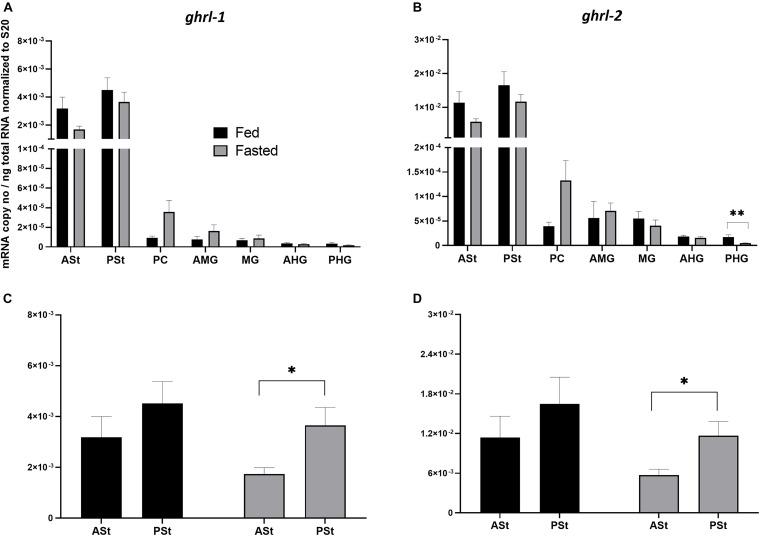
**(A,B)** Comparison of mRNA expression levels of **(A)** *ghrl-1* and **(B)** *ghrl-2* between fed (control) and 4-day fasted groups along the GIT. **(A)** Data are presented as mean ± SE [*n* = 12 per group, except for ASt fasted, PSt fed, PC fed, AMG fed, MG fed, AHG fed, and PHG fasted (*n* = 11), and PC fasted (*n* = 10)] of the normalized expression using the reference gene *s20*. **(B)** Data are presented as mean ± SE [*n* = 12 per group, except for ASt fasted, PSt fed, PC fed and fasted, AMG fed and PHG fasted (*n* = 11), and AMG fed (*n* = 10)] of the normalized expression using the reference gene *s20*. Statistical analysis: unpaired *t*-test. **(C,D)** Comparison of mRNA expression levels between the two stomach segments (ASt and PSt) and the two groups (control and 4-days fasted) for **(C)** *ghrl-1* and **(D)** *ghrl-2*. Two-way ANOVA showed a significant effect of stomach segments [*F*(1, 41) = 9.753; *p* < 0.01] but not presence/absence of food [*F*(1, 41) = 0.3178; *p* > 0.05] for *ghrl-1*; a significant effect of stomach segments [*F*(1, 42) = 9.295; *p* < 0.01] but not the presence/absence of food [*F*(1, 42) = 0.2875; *p* > 0.05] for *ghrl-2*. A Sidak’s multiple comparisons test was used to assess for specific pair-wise differences. ASt, anterior stomach; PSt, posterior stomach; PC, pyloric ceca; AMG, anterior midgut; MG, midgut; AHG, anterior hindgut; PHG, posterior hindgut.

The analysis of *ghrl* mRNA expression levels along the GIT segments of Atlantic salmon showed a high and similar expression trend for both splice variants (Figures 2A,B), with high expression in the two stomach segments (ASt and PSt) and residual or absent expression along the other GIT segments. *ghrl-2* (Figure 2B) was much more abundant than *ghrl-1* (Figure 2A). The comparison between fed and fasted groups revealed a tendency of downregulation for fasted group in the stomach sections, and a statistical difference was only found in the PHG for *ghrl-2* (*p* < 0.01). To evaluate different mRNA expression levels in the two stomach segments between the two groups (Figures 2C,D), a two-way ANOVA test (referring to presence/absence of food and stomach segments as variables) was used, and the results showed a significant effect of stomach segment in the fasted group for both splice variants.

### Correlation Analysis Between mRNA Expression and GIT Sections Content

**FIGURE 3:**
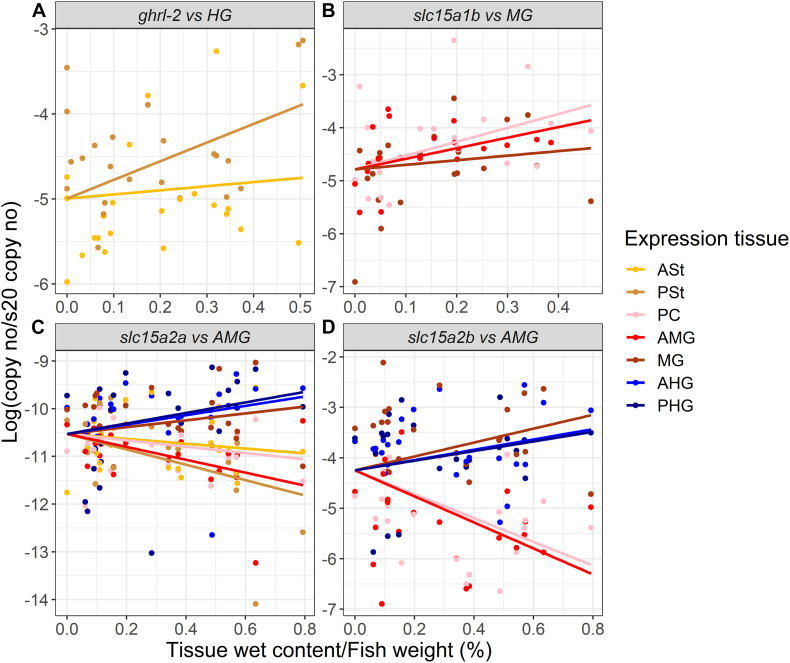
Estimated correlation (solid line) between **(A)** *ghrl-2*, **(B)** *slc15a1b*, **(C)** *slc15a2a*, and **(D)** *slc15a2b* mRNA expression and the gastrointestinal tract sections digesta/inner content. Raw data are represented by dots (*n* = 24, except when outliers were removed as described for Figures 1, 2). ASt, anterior stomach; PSt, posterior stomach; PC, pyloric ceca; AMG, anterior midgut; MG, midgut; AHG, anterior hindgut; PHG, posterior hindgut. Refer to Supplementary Table 1 for detailed information.

When exploring how the presence/absence of food (nutrients) in the different sections of the GIT might affect the expression of our target genes (restricted to the tissue with relevant expression levels), *ghrl-1* and *ghrl-2* (two independent variables) were found highly correlated (Pearson’s correlation > 0.95), and the analysis was therefore performed only for one splicing variant (Supplementary Figure 2). *ghrl* mRNA expression in the stomach is mainly explained by the HG content and resulted in a statistically significant correlation (*p* < 0.01) between expression in the PSt and the HG content (Figure 3A and Supplementary Table 1).

*slc15a1a* and *slc15a1b* mRNA expressions were also found highly correlated (Pearson’s correlation > 0.95), and therefore, the analysis was carried out using the most abundant paralog, *slc15a1b* (Supplementary Figure 2). The mRNA expression of *slc15a1b* in the PC, AMG, and MG (GIT regions with highest expression levels, see Figure 1B) were better explained by the MG inner content, with a statistically significant correlation between MG inner content and PC (*p* < 0.01) and AMG (*p* < 0.5) *slc15a1b* mRNA expression (Figure 3B and Supplementary Table 1). The GIT mRNA expression levels of Atlantic salmon *slc15a2a* and *slc15a2b* were better explained by the AMG content. *slc15a2a* mRNA levels in the PSt (*p* < 0.001), AMG (*p* < 0.01), MG (*p* < 0.05), and AHG (*p* < 0.05) were significantly correlated with the AMG content (Figure 3C). *slc15a2b* mRNA expression was found in the AMG and PC significantly highly correlated (*p* < 0.001) with the AMG inner content, and expression in the MG was also significantly (*p* < 0.05) correlated with the MG content (Figure 3D and Supplementary Table 1). Interestingly, mRNA expression of *slc15a2b* was negatively correlated (Pearson’s correlation > 0.85) with the *ghrl* mRNA expression (Supplementary Figure 2).

## Discussion

Sensing of nutrient luminal composition and quantity provides the vertebrate GIT the ability to modulate the response to feeding or a meal depending on chemical, physical, mechanical, and external signals (Rønnestad et al., 2014; Daniel and Zietek, 2015; Zietek and Daniel, 2015). Therefore, the aim of our study was to investigate the GIT response to a 4-days induced fasting in Atlantic salmon. The study was based on the mRNA expression levels analysis of several gastrointestinal genes modulated by nutrient status in the GIT, i.e., *slc15a1a*, *slc15a1b*, *slc15a2a*, and *slc15a2b* (as marker of intestinal uptake of di- and tripeptides and peptide chemosensor), and *ghrl-1* and *ghrl-2* (as marker of enteroendocrine hormone production and gut–brain signaling axis). Furthermore, the analysis also explored if fish size and condition affected evacuation, assessed as gastrointestinal fullness (digesta content/fish weight %) after 4 days of fasting. Additionally, the mRNA expression levels of our target genes were correlated with the content of each GIT compartment.

There were no differences in length and weight between control and fasted salmon, whereas *K* was significantly lower in the fasted fish, indicating a slightly leaner fish with less fat, which could be expected after 4 days without feed. However, the analysis of the gastrointestinal fullness shows residual digesta in all the GIT compartments of fasted salmon (except in stomach), demonstrating that 4 days of fasting is not enough for a complete evacuation, although at this time, the majority of the digestible nutrients in the feeds could be expected to be absorbed, and the remaining content likely would be nondigestible feed ingredients, microbiota, ions, and water. This is supported by a previous study on Atlantic salmon that showed that 48 h were enough to completely empty stomach and the anterior part of the intestine, and most of the dry matter was only present in the HG at that time point (Aas et al., 2017). Thus, the GIT yet had not been totally cleared for indigestible remnants 4 days after the last meal.

The transcriptional analysis involved, first, the peptide transporters. Pept1 is an important intestinal transporter of di- and tripeptides characterized by an expression largely varying during ontogeny, in response to nutritional status (for, e.g., food limitation/deprivation and fasting/refeeding), dietary amino acids, environmental conditions, and under certain disease state (such as gut inflammation) (Amberg et al., 2008; Bakke et al., 2010; Verri et al., 2010, 2017; Bucking and Schulte, 2012; Romano et al., 2014). The rostro-caudal gene expression analysis along the GIT (high expression in PC, AMG, and MG) and the expression ratio levels of both paralogous genes (*slc15a1b* mRNA expression levels largely exceeding those of *slc15a1a*) are in line with previous studies on Atlantic salmon (Rønnestad et al., 2010; Gomes et al., 2020) and other salmonids such as rainbow trout (Ostaszewska et al., 2010; Kamalam et al., 2013). In our study, 4 days of fasting induced a moderate tendency of downregulation of both *slc15a1* paralogous genes along the GIT, with statistical differences in the PC for *slc15a1a* and in PC and MG for *slc15a1b*. In more detail, 4 days of fasting induced a 24% reduction in mRNA expression levels along the GIT for *slc15a1a*, with a peak of a 42% reduction in PC. Conversely, for *slc15a1b*, fasting induced a stronger effect, with a reduction of 42% along the GIT (peaks of ca. 55 and 45% of mRNA expression levels in PC and MG, respectively). Our analysis is in agreement with previous studies showing that fasting induced downregulation of mRNA expression in teleost species, which is the opposite of what happens in mammals (Thamotharan et al., 1998; Ogihara et al., 1999), and this most likely represents a different strategy of adaptation to food deprivation/limitation between ectotherms and endotherms. Six days of fasting induced as much as ca. 70% reduction in intestinal *slc15a1b* mRNA levels of Atlantic salmon (Rønnestad et al., 2010). Furthermore, in Nile tilapia, 1 week of food deprivation induced ca. 50% reduction of intestinal *slc15a1a* mRNA levels, although only 15% reduction was recorded for *slc15a1b* mRNA (Huang et al., 2015); in European seabass, 5 weeks of food deprivation induced ca. 70% reduction of intestinal *slc15a1b* mRNA levels, with a beginning of the downregulation already detected after 4 days of fasting (Terova et al., 2009); and in zebrafish, a progressive downregulation of *slc15a1b* mRNA levels was recorded during 5-days induced fasting (Koven and Schulte, 2012). In our analysis, the effects of fasting on the gene expression variation were not evaluated in the complete GIT but, rather, in specific gut compartments and thus identifying PC and MG as the most responsive compartments to fasting. Furthermore, for the first time in Atlantic salmon, *slc15a1a* is shown to respond to fasting with lowering mRNA expression levels, confirming the high flexibility of both transporters in the context of gut physiology and their ability to respond to different luminal conditions and signals, as previously shown in Mozambique tilapia (Con et al., 2019), in Nile tilapia (Hallali et al., 2018), and in European sea bass (Kokou et al., 2019). Based on correlation analysis, moreover, the *slc15a1* expression in PC and AMG appeared to be associated with the residual digesta content of the MG, suggesting high sensitivity of the peptide transporter to external stimuli.

Pept2 is another di- and tripeptide transporter member of the SLC15 family, and together with Pept1 in Atlantic salmon, the paralogs originate in the salmonid-specific WGD events (Davidson et al., 2010; Berthelot et al., 2014; Macqueen and Johnston, 2014). The Pept2-like proteins seems to be encoded by different paralogous genes. Our study, which is the first report of *slc15a2* in Atlantic salmon, shows higher expression of *slc15a2b* (comparable mRNA levels to *slc15a1b*), with low or absent localization in stomach segments, intermediate expression in PC and AMG, and higher expression levels in the distal intestinal segments. In contrast, *slc15a2a*, compared to b-type paralog (but also compared to both *slc15a1* types), shows a very low and constant mRNA expression levels along the GIT, suggesting a minor role in intestinal uptake of di- and tripeptides. Our data are in line with previous detections of *slc15a2* in the intestine (Romano et al., 2006; Ostaszewska et al., 2018) and, in particular, in the distal intestinal segments, as reported in Nile tilapia (Huang et al., 2015), in juvenile turbot (*Scophthalamus maximus*) (Xu et al., 2016), and in Mozambique tilapia (Con et al., 2019), suggesting a downstream concerted integration of Pept2 with Pept1 in the functionality of gut physiology, in order to completely absorb di- and tripeptides from the intestinal lumen (Smith et al., 2013b; Bisesi et al., 2015). However, in contrast to zebrafish (Tian et al., 2015), 4 days of induced fasting did not seem to affect *slc15a2* mRNA levels (for both types) in Atlantic salmon, except for a higher expression of the fasted group in AMG for *slc15a2b*, and this difference might be due to different teleost species studied (Cypriniformes vs. Salmoniformes family representatives). Furthermore, the utilization of more time points, as done in Tian’s work, should be used to better understand the influence of fasting on the *slc15a2* activity. However, the presence of residual digesta content in the GIT sections might have played a role in the absence of an effect exerted by fasting, as suggested by the evidence that both *slc15a2* mRNA expression detected in the segments with the highest levels show a significant correlation with residual digesta in AMG.

Next, *ghrl* mRNA expression was analyzed, a peptide hormone mainly secreted by the stomach. In Atlantic salmon, the two *ghrl* transcripts (*ghrl-1* and *ghrl-2*) show, as expected, the same tissue distribution along the GIT, with high expression in the two stomach segments (ASt and PSt), and low or absent localization in the remaining distal tracts. Furthermore, higher mRNA expression levels of the *ghrl-2* splice variant were found, although this does not agree with comparable mRNA levels recorded by Murashita et al. (2009). Interestingly, both splice variants showed an mRNA expression affected by localization in the stomach (ASt and PSt) in the fasted group, indicating the posterior part as a major site of production. Fasting did not seem to affect *ghrl* mRNA expression in the stomach segments, and only a moderate, and not significant, downregulation trend was observed. This result does not support an orexigenic function by Ghrl as seen in mammals (Patton and Mistlberger, 2013) and in many teleost fish species, such as in 7-days fasted goldfish (Unniappan et al., 2002), Nile tilapia (Schwandt et al., 2010), and gibel carp (*Carassius auratus gibelio*) (Zhou et al., 2016). However, in Salmoniformes, contradictory results about Ghrl role are widespread. In rainbow trout, central Ghrl injections and long-term peripheral treatments determined an anorexigenic response (Jönsson et al., 2010), whereas Velasco et al. (2016a, b) observed an orexigenic effect. In Atlantic salmon, plasma Ghrl levels were higher in fasted fish compared to fed fish after 2 days, but after 14 days, there were no differences between treatments (Hevrøy et al., 2011), whereas 6 days of fasting induced increased *ghrl-1* mRNA expression levels (Murashita et al., 2009). In our analysis, no correlation was found between stomach content and *ghrl* mRNA expression levels (stomach is the compartment where *ghrl* is mostly expressed) but, rather, between mRNA expression in the PSt and inner content in HG. A previous study indicated a correlation between a complete empty stomach with an increase in only one orexigenic factor (i.e., *agrp1* mRNA expression levels) in the hypothalamus of 3 days of induced fasting in Atlantic salmon (Kalananthan et al., 2020b), supporting the hypothesis that 4 days of fasting are not sufficient to initiate a significant response. Thus, it is still uncertain how the multiple gastric and intestinal signaling factors responds to fasting with different durations. For this reason, a further analysis using more time points should be performed. Moreover, different parameters, such as condition factor, duration of fasting, and stress, will also affect the physiological response of the fish, and for these reasons, further research is required, focusing also on the link between sensory systems like peptide transporters with hormones to better understand their functional role.

In conclusion, our analysis provides a description of multiple genes related to digestive function and with links to appetite control to elucidate their role in the complex mechanism of intestinal luminal sensing in Atlantic salmon. The main changes were related to the expression of *slc15a1* paralogous genes with tract specificity, whereas the ghrelin variants were not affected by changes; this suggests a separation of responses between sensing/transport pathways and hormonal pathways. Overall, the marginal effects on mRNA expression levels of the investigated genes suggest that 4 days of fasting might be a too short period to initiate large gastrointestinal transcriptomic responses in Atlantic salmon.

## Data Availability Statement

The raw data supporting the conclusions of this article will be made available by the authors, without undue reservation.

## Ethics Statement

Ethical review and approval was not required for the animal study because the experiment and sampling were conducted in accordance with Norwegian Animal Research Authority regulations and were approved by local representative of Animal Welfare at the Department of Biological Sciences, University of Bergen (Norway).

## Author Contributions

IR, SH, FL, AG, and TK conceived the study. TK executed the experiment. TK, IR, SH, and FL performed the sampling. GDV and FL performed preparatory lab work and RT-qPCR analysis. AG, FL, and GDV performed statistical analysis and related graphs. GDV, AB, and TV prepared the first draft of the manuscript. All authors contributed to the interpretation of the data, writing of the manuscript, and reading and approving the submitted version.

## Conflict of Interest

The authors declare that the research was conducted in the absence of any commercial or financial relationships that could be construed as a potential conflict of interest.
