# Supplementary material for: Effects of Short-Term Fasting on mRNA Expression of Ghrelin and the Peptide Transporters PepT1 and 2 in Atlantic Salmon (Salmo salar)
Source: Front Physiol. 2021 Jun 21;12:666670. doi: 10.3389/fphys.2021.666670 (PMC8255630; doi:10.3389/fphys.2021.666670)
Supplement: Supplementary file 1 [file Data_Sheet_1.docx]

**Supplementary Material**

**Supplementary Table 1.** Results from the generalized linear model (GLM) for log transformed mRNA expression levels *versus* gastrointestinal tract sections wet weight digesta content (*n* = 24 per group; see Fig.1 and Fig.2 for outliers’ information). The results from the best model are presented (selection based on AIC). Estimates, 95% confidence interval (CI) and p-values are given. (* = p < 0.05; ** = p < 0.01; *** = p < 0.0001).

| ***ghrl-2* ̴ hindgut inner content** | | | | |
| --- | --- | --- | --- | --- |
|  | **Estimate** | **95 % CI** | ***p*-value** |  |
| *(Intercept)* | -5.00 | 0.29 |  |  |
| Anterior stomach | 0.48 | 1.43 | 0.509 |  |
| Posterior stomach | 2.20 | 1.45 | 0.005 | ****** |
| ***slc15a1b* ̴ midgut inner content** | | | | |
| *(Intercept)* | -4.79 | 0.24 |  |  |
| Pyloric caeca | 2.61 | 1.56 | 0.002 | ****** |
| Anterior midgut | 2.00 | 1.89 | 0.043 | ***** |
| Midgut | 0.86 | 1.68 | 0.317 |  |
| ***slc15a2a* ̴ anterior midgut inner content** | | | | |
| *(Intercept)* | -10.53 | 0.19 |  |  |
| Anterior stomach | -0.51 | 0.86 | 0.248 |  |
| Posterior stomach | -1.61 | 0.88 | <0.001 | ******* |
| Pyloric caeca | -0.66 | 0.88 | 0.143 |  |
| Anterior midgut | -1.35 | 0.89 | 0.003 | ****** |
| Midgut | 0.72 | 0.86 | 0.103 |  |
| Anterior hindgut | 0.98 | 0.86 | 0.026 | ***** |
| Posterior hindgut | 1.10 | 0.86 | 0.013 | ***** |
| ***slc15a2b* ̴ anterior midgut inner content** | | | | |
| *(Intercept)* | -4.25 | 0.26 |  |  |
| Pyloric caeca | -2.36 | 1.06 | < 0.001 | ******* |
| Anterior midgut | -2.58 | 1.10 | < 0.001 | ******* |
| Midgut | 1.38 | 1.06 | 0.013 | ***** |
| Anterior hindgut | 1.02 | 1.06 | 0.063 |  |
| Posterior hindgut | 0.95 | 1.12 | 0.099 |  |

**
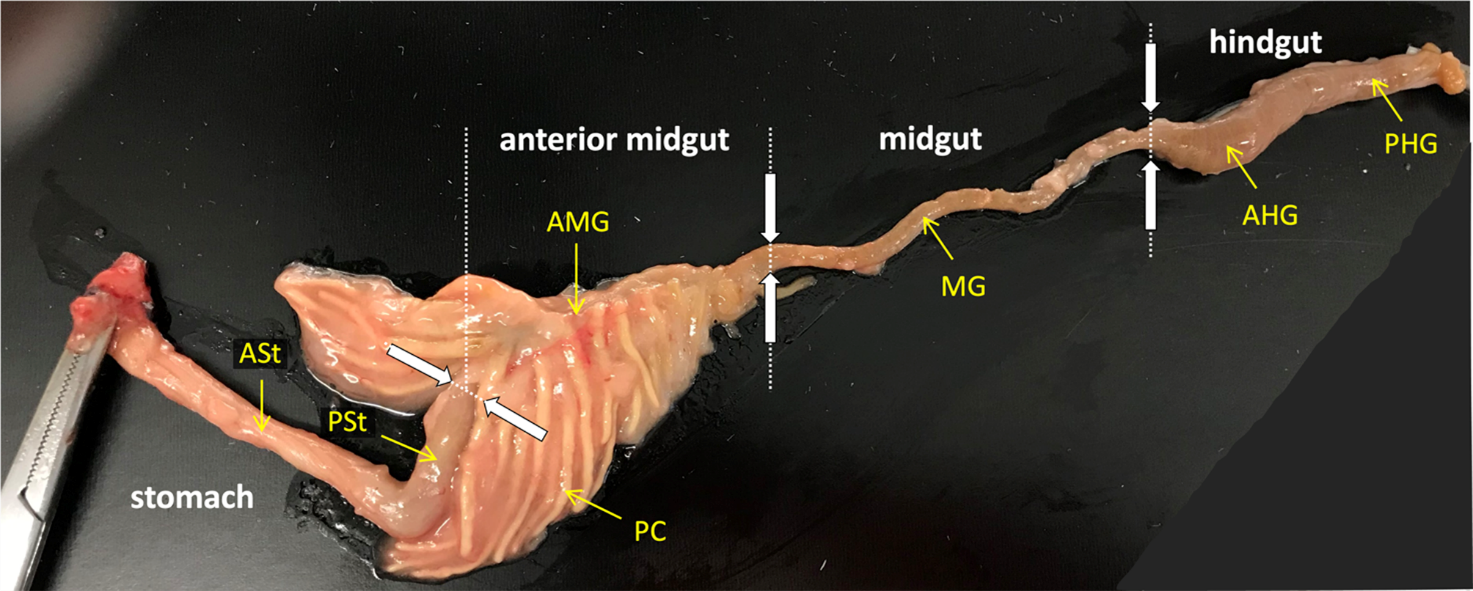
**

**Supplementary Fig. 1.** Collection of samples from the Atlantic salmon gastrointestinal tract (GIT). White arrows represent the position where the surgical clamps were used to divide the gut into four distinctive compartments (stomach. anterior midgut. midgut and hindgut). Tissue samples for analysis of mRNA expression of selected genes were taken from locations indicated by yellow arrows: anterior stomach (ASt). posterior stomach (PSt). pyloric caeca (PC). anterior midgut (AMG). midgut (MG). anterior hindgut (AHG) and posterior hindgut (PHG). Photo by T. Kalananthan.


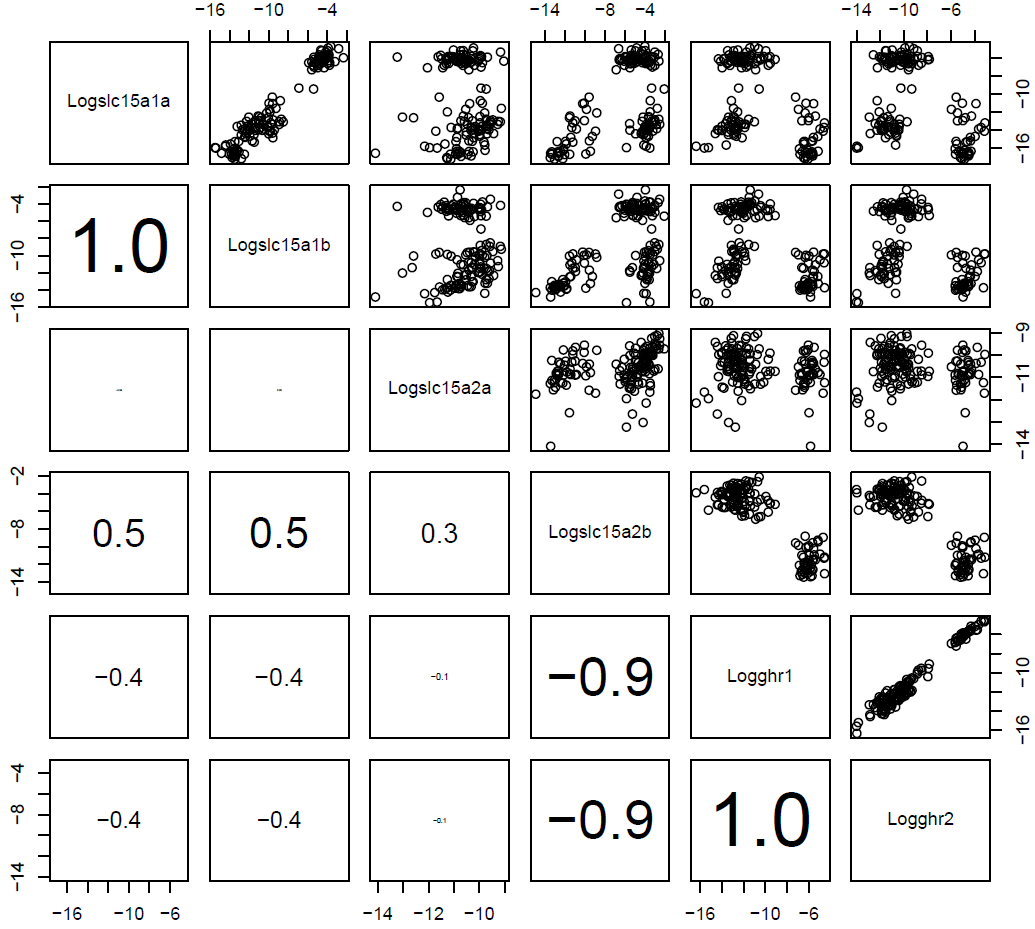


**Supplementary Fig. 2.** Multi‐panel scatterplot of log mRNA expression data in all gastrointestinal tract (*n* = 24). The upper/right panels show pairwise scatterplots between each variable and the lower/left panels contain Pearson correlation coefficients. The font size of the correlation coefficient is proportional to its value.
